# Bocavirus Viremia and Hepatitis in an Immunocompetent Child

**DOI:** 10.4274/balkanmedj.2015.1492

**Published:** 2017-05-15

**Authors:** Zeliha Haytoğlu, Oğuz Canan

**Affiliations:** 1 Department of Pediatrics, Çukurova University School of Medicine, Adana, Turkey; 2 Department of Pediatrics, Division of Pediatric Gastroenterology, Başkent University School of Medicine, Adana, Turkey

**Keywords:** Bocavirus, hepatitis, viremia, immunocompetent child

## Abstract

**Background:**

So far, many studies have shown that Human Bocavirus ( HBoV) is the main pathogen of the respiratory tract. Until now, there is no study that proves the association between HBoV and hepatitis. HBoV viremia/DNAemia has been associated closely with acute primary infection and moderate-to-severe illness but, more detailed clinical data about HBoV dissemination are still unavailable.

**Case Report:**

Here we report a 2-years-5-months-old girl suffering from respiratory distress and heptitis followed in our intensive care unit. HBoV was detected in our patients nose and throat swabs concurrent with whole blood sample by positive polymerase chain reactions. After a through investigation no causative agent other than HBoV viremia was found.

**Conclusion:**

Human Bocavirus viremia with high viral loads may be associated with hepatitis.

Human bocavirus (HBoV) is a newly described human pathogen that has been frequently associated with upper and lower respiratory tract infections. The reported prevalance of the virus has been ranging from 2% to 19% (1,2). HBoV infections are frequently detected in <2-year-old children often with other respiratory viruses (3,4). The clinical manifestatons of HBoV respiratory tract infection have ranged from mild upper respiratory disease (3,4) to severe life-threatening pneumonia (2,5). The direct impact of HBoV infection of the respiaratory tract is often difficult to assess because of its frequent detection in asymptomatic children and coinfection with other respiratory viruses in symptomatic children (4,6). A causal link between HBoV and respiratory disease has been reported but the exact clinical characteristics await determination. In children HBoV may cause more severe clininical conditions such as encephalitis and life-threatening complications (7,8). Although previously bocavirus associated hepatitis in an immuncompromised patient was mentioned in a single case report (9) here, we confirmed HBoV infection in an immunocompetent 2-years-5-months-old girl with hypoxia, cough, fever demonstrating respiratory tract infection along with hepatitis. To the best of our knowledge this is the first reported case of possible bocavirus associated hepatitis in an immunocompetent child.

## CASE PRESENTATION

A previously healthy 2-year-5-month-old girl was admitted to our hospital with complaints of fever, vomiting, abdominal pain and runny nose for the previous 5 days. No diarrhoea or any rash was present. Her past medical history was unremarkable. She had never been hospitalized previously. There was no history of any medication usage or toxin exposure and no travel history. Because of respiratory distress, manifested by nasal flaring, intercostal, subcostal and suprasternal retractions, a respiratory rate of 80 breaths per minute and cyanosis, heated humidified high-flow nasal cannula (HFNC) therapy was used in our intensive care unit to reduce the work of breathing. At the time of admission, her laboratory findings were as follows; white blood cell count 13x10^9^/L, absolute neutrophil count 2500, haemoglobin 9.2 g/dL platelet counts 330x10^9^/L, C-reactive protein level 47.8 mg/dL, alanine aminotransferase level 4113 U/L, aspartate aminotransferase level 7055 U/L, total biluribin 0.8 mg/dL, direct biluribin 0.6 mg/dL, activated partial thromboplastin time 31.6 seconds, prothrombin time 24.9 seconds, international correction rate 2.38, albumin 25 g/L. The gamma-glutamyl transpeptidase and alkaline phosphatase were normal. Laboratory and clinical findings were consistent with hepatitis and bronchiolitis. Serological testing were as follows: mycoplasma IgM(-), IgG(-), HBsAg(-), AntiHbs(+), HAV IgM(-), HAV IgG(-), AntiHCV(-), AntiHIV(-), EBV IgM(-), EBV IgG(-), CMV IgM(-), CMV IgG(-), human herpes virus 6 (-) herpes IgM(-) herpes IgG(-). Varicella IgM(-), IgG(+). Lyme IgM(-), IgG(-) Rose Bengal and Wright tests for salmonella and brucellosis serology was negative. Blood culture was negative for bacterial infections. Stool and blood specimens were negative for enteroviral infections. No spesific causative agent of hepatitis was found after a through investigation. At the time of admission, a diagnostic PCR analysis of a nasopharyngeal aspirate (NPA) swab was performed (LightCyler 2.0; Roche, Germany). HBoV, human coronavirus group 1 and group 2, human metapneumovirus, influenza virus types A and B, and respiratory syncytial virus, adenovirus, parainfluenza virus 1-4 and Rhinovirus were searched for the diagnostic panel. NPA was positive for only HBoV, PCR analysis was negative for all other viruses. HBoV viral load was detected 8x106 copies per ml serum in blood samples concurrent with the NPA. The patient’s respiratory status was improved over three days to normal. The hepatitis did not become severe and with supportive medical treatment the patient was discharged four days after admission. The written informed consent for participating in this report was obtained from the parents of the child.

## DISCUSSION

HBoV has been determined in patients with respiratory infections but its association with hepatitis has been shown only by one report. A case report from Finland in 2008 described an immunodeficient six-month-old boy without respiratory symptoms with hepatitis (9). HBoV DNA was identified from the NPA and blood of their patient by PCR, and diagnosis of acute primary HBoV infection was confirmed by specific IgM positivity in serum, and a fourfold rise of IgG antibody levels in paired sera. Here we described an immunocompetent child with HBoV infection with clinical hepatitis and respiratory symptoms but without other concomitant viruses. The sole detected pathogen was the HboV, both in the NPA and blood of our patient. Respiratory infections due to HBoV are systemic and can be diagnosed serologically, but in our case serological diagnosis of HBoV was not done because these tests were not available at our institution. Although diagnostic serology is helpful for disclosing HBoV infection with disease, some studies suggest that the detection of a high viral load of HBoV genomic DNA in blood may be useful in establishing the diagnosis of HBoV infection with disease (10), and one study also suggested that if HBOV viremia occurs it can disseminate to other parts of the body (1).

When a sole viral finding in the respiratory tract is made, HBoV infection usually occurs with viremia and high viral loads and is often accompanied by the evidence of specific IgM and IgG antibodies (1,10).

In light of these findings we can conclude that the high HBoV viral load in the blood sample of our patient can be considered as evidence of acute HBoV disease and hepatitis may be the clinical result of the dissemination of HBoV viremia.

There is limited data about the link between HBoV and hepatitis. To date, the pathogenesis of HBoV infection has not been fully elucidated. HBoV can be cultured only in differentiated human airway epithelial cells and no animal model is available. We detected HBoV as a sole pathogen both in blood and NPA samples. There are confusing data about the disease severity of single HBoV infections compared with HBoV co-infections in respiratory syndromes. Some authors suggest a more severe clinical picture with a high blood load with mono-infection (1,10).

Although the definitive diagnosis of HBoV hepatitis requires the demonstration of the virus in a liver biopsy specimen, in our case a liver biopsy was not done. The diagnosis was made based upon an HBoV viremia and a compatible clinical picture with the exclusion of other causes. The findings strongly suggest the HBoV as a causative agent. The true HBoV pathogenesis is not yet fully understood. Also, the link between HBoV and hepatitits requires supporting evidence. Tropism of HBoV to the hepatic tissue can be studied further. To the best of our knowledge, this is the first report that demonstrates HBov viral load in both nasal and blood samples from an immunocompetent child with hepatitis and respiratory infection but without mixed infections with other viruses, thus supporting the hypothesis that HBoV may cause hepatitis.
